# Pseudo-Ductility, Morphology and Fractography Resulting from the Synergistic Effect of CaCO_3_ and Bentonite in HDPE Polymer Nano Composite

**DOI:** 10.3390/ma13153333

**Published:** 2020-07-27

**Authors:** Tauseef Ahmed, Hamdan H. Ya, Rehan Khan, Abdul Munir Hidayat Syah Lubis, Shuhaimi Mahadzir

**Affiliations:** 1 Mechanical Engineering Department, Universiti Teknologi Petronas, Seri Iskandar, Perak 32610, Malaysia; Muhammad_15001294@utp.edu.my; 2 Department of Mechanical Engineering Technology, Universiti Teknikal Malaysia, Melaka 76100, Malaysia; munir@utem.edu.my; 3 Chemical Engineering Department, Universiti Teknologi Petronas, Seri Iskandar, Perak 32610, Malaysia; shuham@utp.edu.my

**Keywords:** pseudo-ductility, toughness, hybrid nano composite, fractography

## Abstract

Polymeric materials such as High density polyethylene(HDPE) are ductile in nature, having very low strength. In order to improve strength by non-treated rigid fillers, polymeric materials become extremely brittle. Therefore, this work focuses on achieving pseudo-ductility (high strength and ductility) by using a combination of rigid filler particles (CaCO_3_ and bentonite) instead of a single non-treated rigid filler particle. The results of all tensile-tested (D638 type i) samples signify that the microstructural features and surface properties of rigid nano fillers can render the required pseudo-ductility. The maximum value of tensile strength achieved is 120% of the virgin HDPE, and the value of elongation is retained by 100%. Furthermore, the morphological and fractographic analysis revealed that surfactants are not always going to obtain polymer–filler bonding, but the synergistic effect of filler particles can carry out sufficient bonding for stress transfer. Moreover, pseudo-ductility was achieved by a combination of rigid fillers (bentonite and CaCO_3_) when the content of bentonite dominated as compared to CaCO_3_. Thus, the achievement of pseudo-ductility by the synergistic effect of rigid particles is the significance of this study. Secondly, this combination of filler particles acted as an alternative for the application of surfactant and compatibilizer so that adverse effect on mechanical properties can be avoided.

## 1. Introduction

Pseudo-ductility is the mechanical property of a functional material to possess the ability of brittle (high strength) and ductile material (high value of elongation) at the same time [1]. According to the most accepted theory, it is possible for a material to undergo pseudo-ductile behaviour if the deformation starts before the yield point in a tensile test [2]. Therefore, the outright knowledge of various aspects of tensile testing (a measure of hardness, a measure of onset of viscoelastic deformation, the area under the curve and slope of the stress–strain curve of materials) are of great importance [3]. Thus, pseudo-ductility is of significant importance because it increases the range of engineering and technical application of a material [1].

In order to achieve pseudo-ductility in polymeric material, filler particles of nano meter size are used. This performance of nano filler particles is attributed to the spacious surface area of nano particles, with the dimensions in nanometers. For example, a filler bearing dimensions in nanometers possess surface area, as large as 15–25 m^2^/g [4,5]. Specifically, if a crack is generated around a nano filler it is of very minute dimensions as compared to micro or macro sized filler particles, thus retaining the toughness (ductility) of a polymer [6].

After several studies have been dedicated to the mechanical properties of semi crystalline polymers, it is a widespread conception that the strength and stiffness can be readily improved by adding rigid (inorganic) nano-particles in polymeric materials. It is because rigid inorganic particles generally have a much higher stiffness than polymer matrices [7]. On the other hand, it is established in the work of Laura et al. [8] that ductility can be enhanced more effectively by the application of non-rigid rubber filler particles due to higher compatibility as compared to rigid inorganic nano filler particles. However, rigid fillers can still be regarded as a choice for the toughness or ductility of polymer composite. While they do not increase the toughness as substantially as rubber particles, they enhance the young’s Modulus (E), strength, and hardness much better than rubber particles [7].

However, polymers such as HDPE overtakes to become purposelessly brittle or weakens (low ductility) when reinforced with rigid fillers. This is because (a) inorganic nano fillers generate microstructural flaws as a result of inhomogeneity in polymer matrix [9], and (b) the generation of cavities around rigid filler particle, due to difference of elastic moduli [6]. On the other hand, HDPE acquires ductility (low strength) when reinforced with rubber filler particles. Moreover, if there is an adequate bonding between the polymer–filler interfaces in case of rigid filler, or rubber filler particles, mechanical properties can be improved [4]. Therefore, the following section discusses polymer–filler interface as a separate section of the introduction in Section 1.1.

### 1.1. Polymer/Fillers Interfacial Interaction

A worthwhile point for further discussion of mechanical properties in this work is the consideration of structure–property relationship of rigid CaCO_3_ and bentonite (nano clay) filler particles for polymer–fillers interfacial interaction. In order to achieve an adequate polymer–filler interfacial interaction, the study of Sahin et al. [10] and D’Anna et al. [11] asserts the application of surfactants and compatibilizer, respectively. The results explained in the work of Sahin and his co-worker, show that mechanical properties can be enhanced when there is an effective stress transfer from polymer to filler particles provided that the polymer fillers have compatible interfaces. Moreover, the application of compatibilizer reduces the chances of micro-voids during processing. On the other hand, the study of Zhang et al. [12] disagrees with the application of surfactants. Similarly, Liu et al. [13] mentions, that surface coating may not be always effective. Additionally, Moghbelli et al. explained that compatibilizers can even adversely affect mechanical properties [14].

As previously studied separately, bentonite nano clay and CaCO_3_ nano particles have been favorable for enhancing mechanical properties [4]. Apart from mechanical property, nano clay additionally possesses the property of finer compatibilization. Thus, the thermodynamic compatibilizing effects of the clay platelets can be objectively taken into consideration as an alternative for the application of surfactants [15]. In particular, bentonite nano clay possesses enhanced compatibility with polymeric phases [14]. Thus, by harnessing the synergistic effect of CaCO_3_ and bentonite nano clay particles, polymer–fillers interfaces can be improved without application of surfactants and compatibilizer.

With attention to all aspects discussed, bentonite nano clay in combination with CaCO_3_ can enhance strength by rigidity and enhance ductility by compatibilization. Furthermore, ductility can be induced because matrix ligaments can cavitate around the rigid filler particle (crack bowing) or to push against the well-bonded fillers (crack pinning) [16]. The externally applied energy is used in crack pinning or crack bowing giving ductile (tough) behaviour to the matrix [17].

To the best of our knowledge, recent literature does not contain any study to achieve pseudo-ductility with the application of two non-treated rigid filler particles. Here, we innovatively utilized the synergistic effect of bentonite and CaCO_3_ for mechanical, surface and compatibilization properties by loading into HDPE polymer simultaneously. Moreover, the tensile testing, morphological and fractographic characterization revealed that properties can be enhanced without application of surfactant and compatibilizer for certain cases. EDS (Energy Dispersive Spectroscopy) revealed a homogenous dispersion of filler particle and elemental analysis verified this combination of rigid particles for pseudo-ductility. Thus, HDPE nano composite is noticed to acquire pseudo-ductility (high strength and ductility), as compared to its ductile behaviour in pure state.

## 2. Materials and Methods

### 2.1. Materials

HDPE polymer was supplied in the form of pellets from titanvene Malaysia, with the trade name HD5218EA. It has Melt flow index (MFI) and a nominal density of 18 g/10 min and 0.950 gm/cm^3^, respectively. CaCO_3_ (33295 Calcium carbonate) was supplied in the form of a precipitated nano powder with a density of 2.930 gm/cm^3^, formula Weight of 100.09 and melting point of 800 °C. Sodium bentonite with high montmorillonite content (61.5% by mass) and a cation exchange capacity of 66.5 meq/100 g (determined by methylene blue test) was obtained from Alfa Aesar. 

### 2.2. Composite/Sample Fabrication

**Figure 1:**
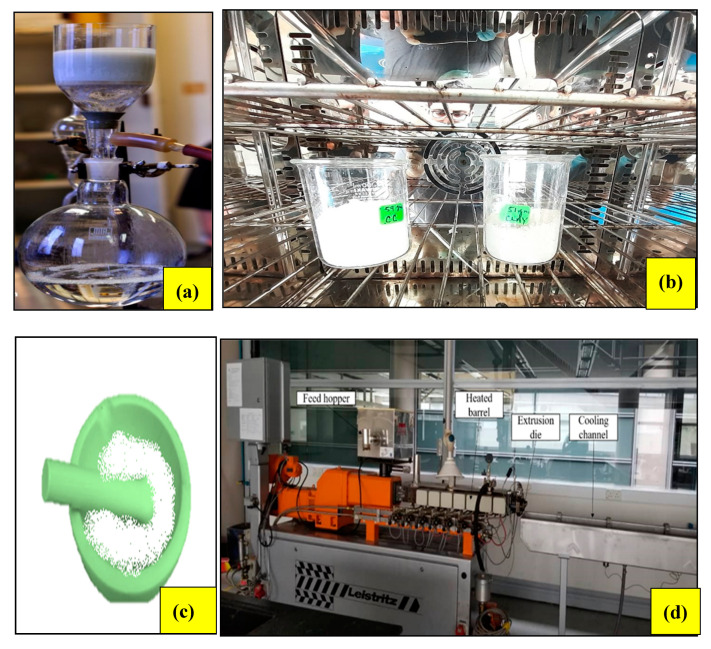
Fabrication process of HDPE polymer nano composite (**a**) vacuum filtration, (**b**) drying in a vacuum oven, (**c**) grinding, (**d**) melt compounding using a twin-screw extruder.

Bentonite and CaCO_3_ are both hydrophilic nano particles and have very low compatibility with the hydrophobic nature of HDPE. Therefore, it becomes imperative to remove water content from non-treated nano particles before feeding into a twin-screw extruder. In order to do so, both the particles were first washed with ethanol as shown in Figure 1a. After that, the vacuum filtration technique was used to separate traces of ethanol from the nano powder as shown in Figure 1b. The filtrate was taken into a vacuum oven for 48 h for drying. The temperature selected was 100 °C. In order to ensure the smaller size of particulates, the dried powder form was grinded by pestle and mortar. All of the aforementioned steps followed the sequence shown in Figure 1a–d.

Formulations selected are on the base of weight percent of fillers and were melt compounded with HDPE resin in a twin-screw extruder. Samples for the tensile test were fabricated separately by using injection moulding. This also enhanced the distribution of filler particles by undergoing melting in combination with filler particles. The temperature for the fabrication of melt compounding of composite and fabrication of samples selected was 120 °C as suggested by the supplier. Moreover, the speed of the screw chosen for melt compounding was 14 r.p.m so as to obtain proper mixing and to avoid unwanted degradation of polymer chains as a result of excessive shear. Furthermore, 4 levels (0,5,10,15) of weight % for three inputs (CaCO_3_, bentonite and HDPE) were chosen by applying the Taguchi design of experiment(DOE) method such that the effect of variable weight % of each component w.r.t other is investigated.

The number of samples prepared is 16 in total (Taguchi DOE), each with a designated label as BC-x/y. This designation is assigned such that B and C indicates bentonite and CaCO_3_, respectively. Following the same order, x indicates the weight % of bentonite while y indicates the weight % of CaCO_3_. For example, a sample labeled as BC-5/10 is the 6th sample having 5% of bentonite and 10% CaCO_3_ by weight. Moreover, some samples contained the weight % of only bentonite or CaCO_3_ such as BC-0/5 or BC-5/0. Such samples are identified as non-hybrid samples while other samples having both the content of nano particles are identified as hybrid samples such as BC-5/10 etc. Furthermore, Taguchi DOE is applied to get samples such as BC-0/5, BC-10/5 and their counterparts such as BC-5/0, BC-5/10, respectively. Specifically, the cumulative weight % (wt %) of these samples are same but the individual wt % of the particles are alternated as clear from numerical values in their labels. By doing so, the effective role of one particle w.r.t other particles can be discussed. 

### 2.3. Methodology

Field Emission Scanning Electron Microscope (SUPRA 55 V P manufactured by Carl Zeiss AG, Oberkochen, Germany) was used for morphological and fractographic analysis). Scanning Electron Microscopy/Energy Dispersive Spectroscopywas used for the analysis of fabrication, and elemental analysis was performed for verification. Samples for morphological characterization were taken from 3 cm from an end of a 16 cm dog bone specimen. For fractographic analysis, the cross-section of the fracture point was taken and then cut cryogenically by a diamond knife at −60 °C in the FESEM facility. The samples were coated with gold layer in sputtering process in order to make them conductive. The deposited gold layer can be observed by performing elemental analysis of the samples. All of the above testing and characterization facilities were provided by the central Analytical Labs, Universiti Teknologi Petronas, Malaysia.

**Figure 2:**
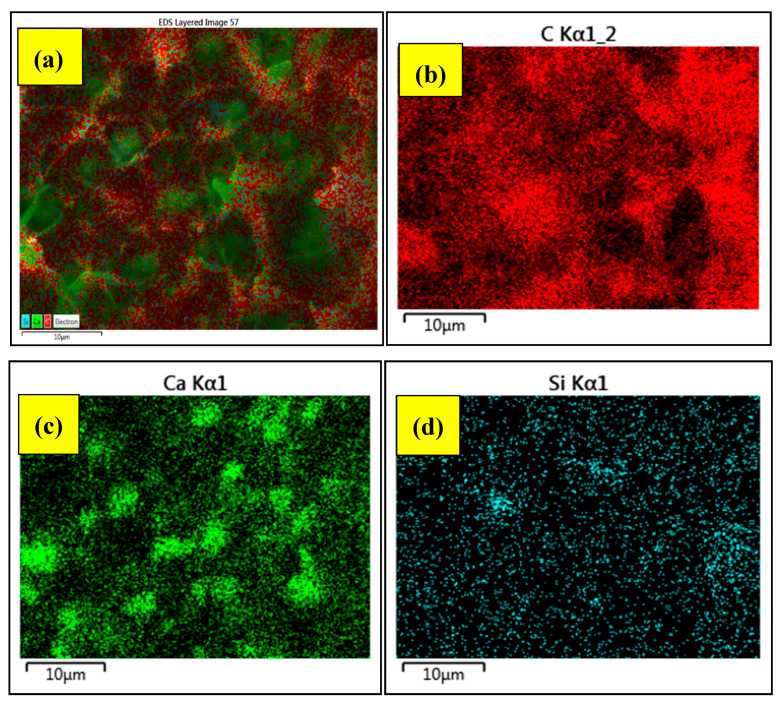
Energy Dispersive Spectroscopy (EDS) mapping for homogeneous dispersion and distribution (**a**) SEM/EDS image of fabricated composite, (**b**) carbon mapping, (**c**) Ca mapping, (**d**) Silicon mapping.

Before, proceeding for Tensile testing, an EDS mapping of fabricated composite is performed, to ensure the best possible distribution of basic element of components [18], as shown in Figure 2. It can be seen that the major components, carbon (C), calcium (Ca), silicon (Si), are homogeneously distributed, so that there are no aggregates and there is no site in the matrix, left unreinforced. The Tensile test is performed according to the ASTM D638 type (i), using a Lloyd Instrument Universal Testing Machine. The Model LR5K is equipped with a 5 kN load cell currently in service in the Lab. According to the test standard, the overall length of the dog bone specimen fabricated is 16 ± 0.50 (mm) and the gauge length is 5 ± 0.50 (mm).

After the test of virgin HDPE polymer, the gauge length reached an elongation of 17 mm. Therefore, 17 mm is assigned as a reference value for comparison with other samples. The test speed chosen for the tensile test is 5 mm/min. Choosing a lowest possible test speed dose not degrade polymer and further allow for a uniform distribution of the externally applied load.

## 3. Results

The pseudo-ductility of the composite is investigated by analyzing and correlating three different mechanical properties, such as tensile strength (TS), Young’s modulus (E), and elongation at break. Moreover, the intent of fabricating a hybrid composite was to achieve a composite that has a higher ultimate tensile strength than the pure polymer and additionally having ductility in agreement with the pure polymer. It is achieved successfully clear by the correlation of tensile strength and elongation at break. Additionally, a comparison was built between the hybrid and non-hybrid samples for an increase in properties to determine the synergistic effects of CaCO_3_ and bentonite. Furthermore, the samples were grouped on the base of similarities in results, due to the weight % of bentonite vs. CaCO_3_. Therefore, group A (GA) comprises the hybrid and non-hybrid samples, bearing more loading of CaCO_3_ as compared to bentonite, while group B (GB) comprises the samples containing more loading of bentonite as compared to CaCO_3_, and group C (GC) has equal comparative weight percent (CW %).

### 3.1. Tensile Strength (σ)

The summary of the tensile testing of GA, GB, and GC is given in Table 1, Table 2 and Table 3, respectively. These tables display the variation in tensile mechanical properties with comparative wt % of CaCO_3_ vs. bentonite added in HDPE. By comparing the data in Table 1 and Table 2, it can be seen that the hybrid samples of GB such as BC-15/5, BC-15/10, BC-10/5 have higher values of strength as compared to their counterparts in GA such as BC-5/15, BC-10/15 and BC-5/10, respectively.

The high value of the tensile strength (TS) of hybrid composite shows that the stress transfer between the rigid fillers and the matrix is well effective. The maximum value of tensile strength achieved is 120 % of the virgin polymer. As displayed in the tables, more than half of the total samples exhibited higher TS as compared to virgin matrix. This includes hybrid samples such as BC-5/15, BC-10/5, BC-15/10, BC-10/10, BC-5/5, 10BC and non-hybrid samples such as BC-5/0 and BC-0/5. It is worth noting for these two non-hybrid samples, that, for the small weight percent of filler particle, TS increases by the physical “chain entanglement” within the matrix. CaCO_3_ and bentonite are the rigid fillers, which impeded the easy sliding of HDPE chains over one another, which increased the TS [19]. This chain entanglement phenomenon is only effective for a smaller weight percent such as 5% (BC-0/5 and BC-5/0).

**Figure 3:**
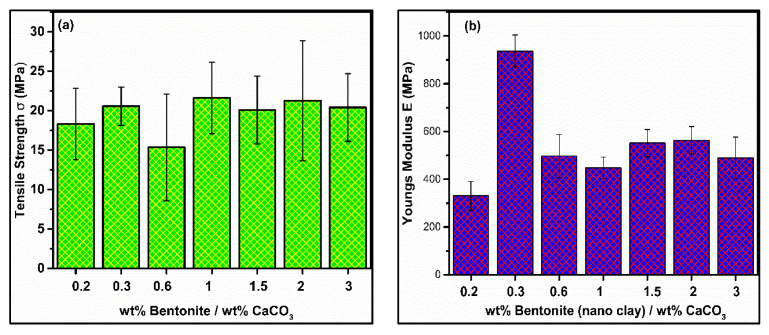

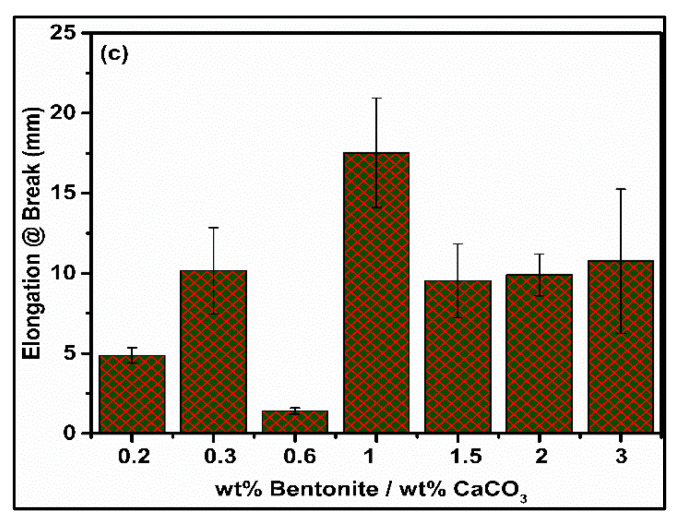
Mechanical properties (**a**), tensile strength Vs comparative weight %(CW%), (**b**) Young’s modulus vs. CW, (**c**) elongation at break vs. CW.

Notwithstanding the tendency for improvement, some samples with exceptionally low tensile strength (less than virgin polymer) are recognized. Specifically, these samples include samples of GA, which are excessively loaded (15–30 wt %), such as BC-0/15, BC-10/15, BC-15/15, BC-5/10, BC-15/0. These deficient results can be attributed to the low values of Equation (1), i.e., when the content of CaCO_3_ dominated bentonite, the agglomerates are not fully disintegrated into finer particles and the melted matrix is more prone to air bubbles. That is to say, the excessive loading of rigid filler increased the chances of air bubbles due to inhomogeneous network density in the HDPE matrix. Thus, the excessive loading of incompatible filler particles effects TS adversely [17]. Therefore, there is no sufficient adhesion between polymer–filler and no sufficient encapsulation of CaCO_3_ particle [20]. The decreasing trend of TS with the lower values of Equation (1) can be depicted as shown in Figure 3a.

On the other hand, 70% of excessively loaded (10–25 wt %) samples in GB show higher TS than virgin polymer, as shown in Table 2. Efficient results shown by GB is due to stress transfer between polymer/filler [21,22] more efficiently as compared to GA and GC due to high bentonite vs. CaCO_3_ ratio, according to Equation (1). This engrossing finding shows that, during processing, bentonite, which is more compatible as compared to CaCO_3_, acted as an agent to change the surface morphology of nano filler to enhance interfacial polymer–filler adherence. This explains the preferential high values of Equation (1) for an increase in TS [15,23].

Comparative Weight % (CW %) = wt % of bentonite (nano clay)/wt % of CaCO_3_ (1)

The homogeneity due to rigid filler particles is reduced in excessively loaded samples of GB and GC due to shear generated by the platelets-like structure of bentonite nano clay during mixing (fabrication). The “knifing” effect during fabrication is introduced by Zhu et al. [24]. The study of Weikang Li [25], although showing the improvement of TS by 36%, the wt % of the filler particles is as low as 5. The required pseudo-ductility cannot be achieved for the lower wt % of the filler particles because the percolation condition cannot be met [26].

The effective synergistic role of bentonite and CaCO_3_ tells us that not always surface treatment, but a careful selection of particles can also effectively transfer the externally applied load between the polymer and the filler even without the application of compatibilizer. The homogeneous distribution of the components, as shown earlier in Figure 2, is the manifestation for this assertion (we also prefer recalling Section 3.5.1 for further explanation) [13,14,27]. Thus, it can be summarized, that for higher values of Equation (1) (high bentonite vs. CaCO_3_), improved TS can be obtained by combination of (a) the compatibilization effect, (b) the knifing effect and (c) the encapsulation of CaCO_3_ filler particles by bentonite. 

### 3.2. Young’s Modulus (E)

Young’s modulus is the slope of the stress–strain curve of a material up to the level of elastic deformation. The fundamental concept of tensile behavior of a material instructs that ideally (perfect bonding between the fillers and the matrix) the more the value of TS, the higher will be value of E. In the same way, the values of E obtained in our work exhibited the results which agreed to the fundamental concept, i.e., the samples with higher values of TS showed higher values of E, such as BC-0/5, which is 788 MPa, and the lower the values of tensile strength for BC-0/0, the lower the value of E, i.e., 596 MPa.

However, cases of observation were noted for some of our samples, which manifests the concept that TS may not be always directly proportional to E. The required low values of E for the samples BC-10/5, BC-15/5, BC-15/10 of GB and BC-5/5 of GC exhibited that load transfer between the three distinct phases of the hybrid composite is well enough to increase the strength, yet the values of E dropped, as shown in Figure 3b. This drop in the values of E is possibly due to the fact that, practically, the permanent deformation started before the yield point or elastic limit [2]. This phenomenon is generally known as “stress softening” [20]. This phenomenon is responsible for the required pseudo-ductility of a material. Reflecting on the values of high elongation at break and high strength states that, in the aforementioned “stress softened” samples, the permanent deformation starts when the strain reaches 1%–2%, irrespective of yield point [2]. The elastic limit lies on the onset of yield point. Therefore, it can be noted to have undergone larger values in each stress-softened sample as compared to non-stress-softened samples.

With no surfactant applied, bentonite and CaCO_3_ have possibly cavitated at the start of deformation (before yield point). This cavitating behaviour offers no constraint to the deforming matrix ligaments, which gives rise to reduced plastic shear resistance around the particles, resulting in a ductile behavior [28]. The load applied during the test was more effective at the voids created at the interfaces rather than at the non-interfacial region. It is firstly because of less probability of voids in the non-interfacial region and secondly due to the better compatibilization effect of bentonite [14].

Moreover, E is the measure of resistance to deformation, which is caused by bonding strength between atoms; therefore, all the non-stress-softened samples having higher values of E are the ones identified with a high strength. The low values of elongation of the non-stress-softened samples, such as -BC-0/5, BC-5/15, BC-5/0, indicate a brittle nature. Such samples possess a high value of strength (brittle) and no ductility, as shown in the Table 1, Table 2 and Table 3.

Observation reveals that all of the stress-softened samples are excessively loaded samples with bentonite and CaCO_3_. The contents of rigid filler particles when enough in amount, are entirely responsible for the toughness jump in the HDPE matrix [28]. Thus, in a tough response, the role of the rubber particles is insignificant and rigid filler particles can be used as an alternative. In the absence of any surfactant, the particles (bentonite+ CaCO_3_) cavitated at the start of deformation with higher values of Equation (1). Thereby the filler particle offered no constraint to the deforming matrix ligaments [20]. It is understandable now, if there is no constraint, the matrix ligaments can elongate, as clear from the high value of elongation of stress-softened samples.

### 3.3. Elongation at Break

The state of literature in the area of mechanical properties points to the need for the correlation of mechanical properties, as there is very low to no study on this subject. Although, loading rigid filler particles reduces elongation in polymeric materials. Yet, the value of elongation retained is, as high as 100%, displayed by BC-5/5 in Table 3. The effective role of synergy can be viewed by increasing trend of elongation with increasing values of Equation (1), as shown in Figure 3c. Thus, our findings disagree with the reduction in elongation with rigid filler particles [29], asserting that it can be retained.

In the case of pseudo-ductility, cavitations start before the yield point. In this particular case, the matrix ligaments are not affected by the rigid filler particles and they are allowed for longer values of elongations. Therefore, all the pseudo-ductile samples can be identified with higher values of elongation, as seen by comparing the data in the Table 1, Table 2 and Table 3. All of the pseudo-ductile samples (BC-5/5, BC-15/5, BC-15/10, BC-10/5) are excessively loaded samples (10–25 wt %). Such a higher weight percent is necessary in order to fulfill the percolation conditions, as explained by Bartczak et al. [26].

It is well established that the elongation of a sample is majorly affected by [15].

- Case 1: Micro-void generated in the matrix during fabrication.
- Case 2: A crack fusing with another crack already generated (by a micro defect or by case 1).

Apart from pseudo-ductile samples, the comparatively higher values of elongation in GB indicates that the effect of case 1 is enhanced by the highly compatible nature of bentonite. Moreover, the fusion of cracks, as in case 2, is avoided by sliding layers of bentonite that can orient perpendicularly to the propagating crack front [13,17]. However, these two factors were not possibly effective for non-hybrid samples such as BC-5/0 and BC-15/15. This behavior can be well attributed to the inadequate breaking of bentonite clusters by the shearing force during the fabrication of BC-5/0 [5] and the excessive loading of fillers in BC-15/15 [20].

Additionally, some infrequent, but important, results for some samples having low loadings of fillers were observed. Samples such as BC-0/10, BC-5/10, and BC-10/10 exhibited exceptionally low elongation even with an exceptionally low tensile strength. Both cases, the excessive voids and the fusion of these voids, could have resulted in this deficient performance. Contrastingly, layered structured bentonite exfoliated well with the oriented and non-oriented (crystalline and non-crystalline) portions of the matrix, avoiding the premature microstructural failure, as in case of the aforementioned samples. For a low loading of fillers, crystalline and non-crystalline parts of the polymer are sometimes separated, and a new morphology of the matrix originates, resulting in different mechanical behaviors (low TS and low elongation of the materials), as noted in the samples mentioned above [26].

Additionally, the values of elongation depend upon the chance of encounters of microstructural flaws with one another during the process of propagation of “neck” in the tensile test. The final fracture for samples is invariably initiated by the agglomeration of fillers at a certain location undergoing “necking.” To illustrate, the elongation of BC-5/15 was 10.16 mm, respectively. That is to say that the more late an encounter of agglomerate occurs during the nonreversible elongation, the later the final fracture takes place.

### 3.4. Load–Extension Curve

**Figure 4:**
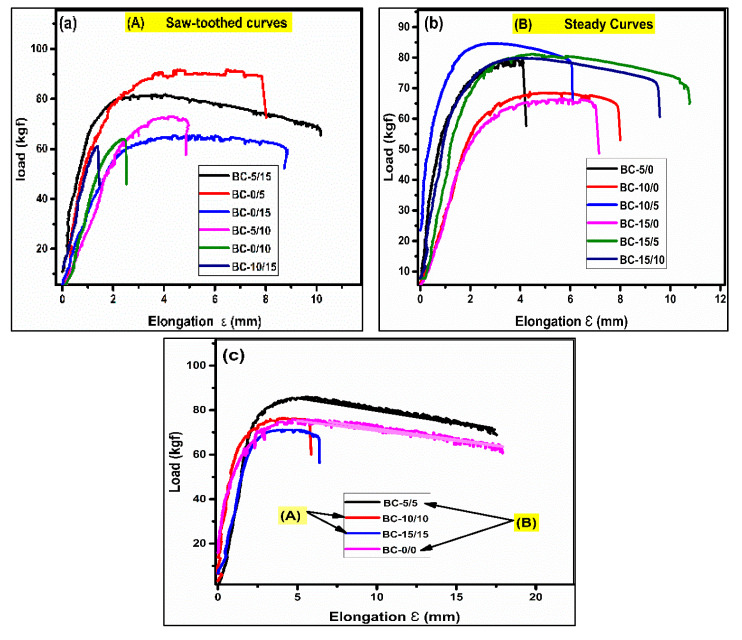
Load extension curve of tensile tested samples: (**a**) group A, (**b**) group B, and (**c**) group C.

**Figure 5:**
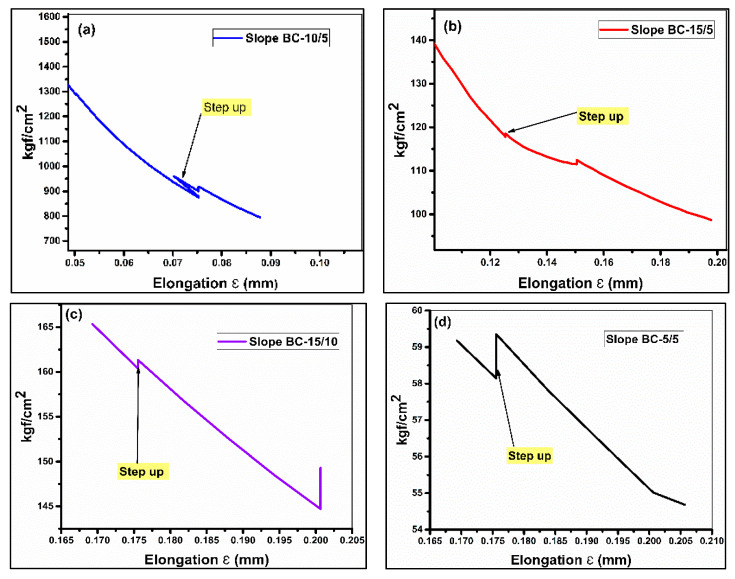
Derivative of load–extension plots of stress-softened samples: (**a**) BC-10/5, (**b**) BC-15/5, (**c**) BC-15/10, and (**d**) BC-5/5.

Analyses of the load–extension curves and its derivative can give information about mechanical properties coupled with the toughening mechanism of the samples as shown in Figure 4 and Figure 5.

The predominant trend of the curve propagation of GA is of a stick and slip manner, as shown in Figure 4a. The maximum value of elongation obtained in GA is of BC-5/15. It is a sample that also stands out in terms of the highest value of E as clear from its slope. Secondly, all the samples of GA can be seen to have undergone an unstable brittle fracture except a stable curve of BC-5/15 [30]. Due to the stick and slip trend, the curves are called as saw–tooth curves. All of the samples of GA are brittle, as is clear from the saw–tooth propagation. The steady curve of BC-5/15 is possibly due to a higher value of elongation or possibly due to the effect of testing condition [12].

On the other hand, the predominant crack propagation in GB is of a stable ductile pattern. All the stress-softened samples can be classified as undergoing a similar stable ductile fracture to that shown by the hybrid samples of GB and BC-5/5 of the GC in Figure 4b,c, respectively. In Figure 4c, a stable ductile curve of BC-0/0 (virgin HDPE) and BC-5/15 is shown. However, the other two samples of GC and indicated non-hybrid samples of GB have identical saw-toothed curves to those of GA [12]. Additionally, saw-toothed curves in each respective group stand out with a smaller area under the curves indicating the lesser values of toughness as compared to samples having stable ductile curve propagation. 

Besides the load–extension curve of the tensile test, the derivative plot of stress–strain curves can give details about the occurrence of toughness. When there is less cavitation in the non-interfacial zones (matrix phase), the stress is concentrated at interfaces between the polymer and the filler. The debonding between the polymer and filler absorbs externally applied energy, which is responsible for the tough response [26].

A derivative plot is constructed on the concept that debonding starts before yielding. Therefore, we analyzed the stress–strain curves in deformations inferior to 2% [2]. In Figure 5, it is noticed that there is a sharp “step-up” of the derivative curve of each stress-softened samples. This kind of sharp increase indicates the toughness jump in the stress-softened samples [31].

### 3.5. Morphological Analysis 

#### 3.5.1. Dispersions Analysis

**Figure 6:**
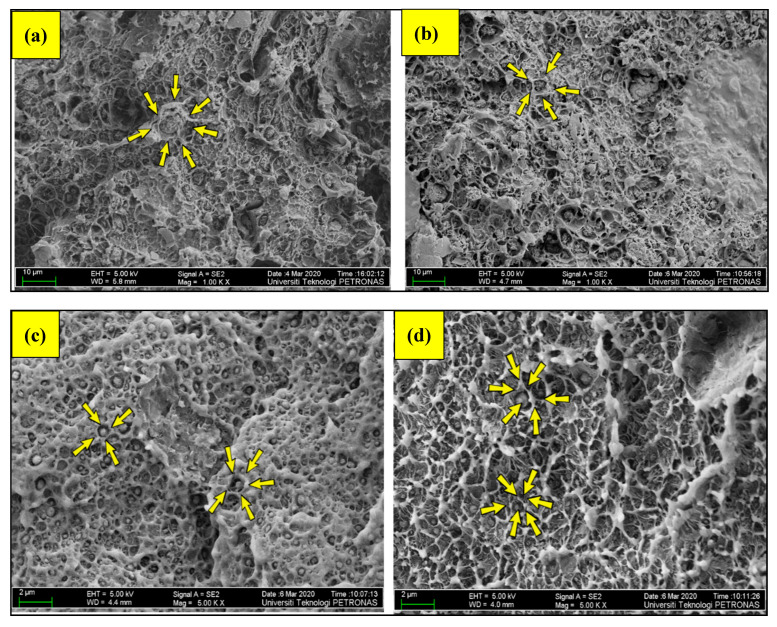
Comparison of morphological patterns: (**a**) BC-5/5 no cavitation around the filler, (**b**) minor cavitation in BC-15/5, and (**c**) noticeable cavitation around the CaCO_3_ fillers in BC-0/15 and (**d**) BC-5/10.

The morphological analysis of the nanocomposite leads to engrossing details, indicating a physio-mechanical phenomenon that enhanced the mechanical properties of HDPE polymer. As is evident from the values of the tensile test, the comparative weight percent of bentonite vs. CaCO_3_ influenced the stress-softening phenomenon; a distinctive shift of patterns is also found in the morphology of GA, GB and GC. In contrast to GB, the results of GC indicates that there is no noticeable difference due to the similar wt % of bentonite. As an illustration in Figure 6, there is an understandable difference of cavitation in samples of different groups, indicated by arrows around the filler particles. Figure 6a is the FESEM image of the sample BC-5/5 holding an equal amount of content of bentonite and nano clay. There is no cavitation around the combination of clay and CaCO_3_ particles owing to the reason that the clay possesses the property of finer compatibilization and bonding to the polymer [14].

As explained in the work of Ruamcharoen et al. [23] and Tiwari et al. [15], the thermodynamic compatibilizing effects of the clay platelets can be objectively taken into consideration for the effective bonding effect with polymer. For sample BC-5/5, which belongs to GC, and all the samples belonging to GC and GB, the filler possessed more or less the same morphological pattern as in Figure 6a,b, provided that mechanical properties were enhanced. Moreover, Figure 6b represents the case of minor voids and cavitation around the filler particles. It indicates compatibility between the polymer and the filler because the particles are adequately bonded to the matrix shown as BC-15/5 in Figure 6b. Thus, the higher values of the TS of GB and GC are due to polymer–filler bonding, as displayed in Table 2.

It is worth noting that all samples belonging to GC and GB displayed morphological patterns that are more or less the same. However, the morphological patterns changed completely when the content of CaCO_3_ particles dominated the composite, as seen from Figure 6c,d, which is BC-0/15 and BC-5/10, respectively, which belongs to GA. This noticeable difference is caused by the extent of inhomogeneity caused by the CaCO_3_ particles.

**Figure 7:**
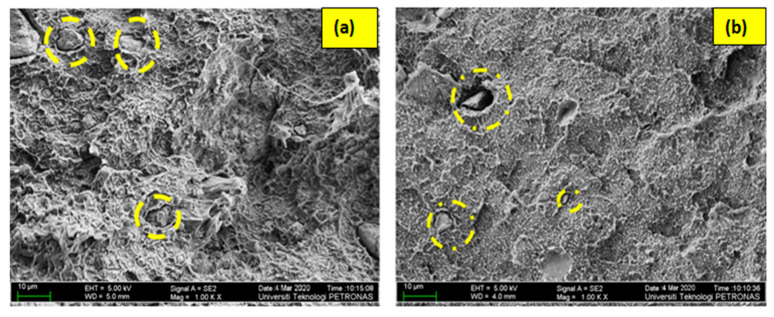
Agglomerated CaCO_3_ particles in samples (**a**) BC-15/15 and (**b**) BC-10/15.

That is to say, the excessive loading of rigid filler increases the chances of air bubbles due to inhomogeneous network density in the HDPE matrix when there is no polymer–filler compatibility [17]. Moreover, the chance of cavitation increases as the weight % of the CaCO_3_ filler particles increases, which is responsible for creating flaws in the composites [32]. This existence of flaws, particularly around the filler particles, hinders the processes of stress transfer between the polymer and fillers, which can be considered the reason for the low tensile strength of samples of GA. As discussed earlier, (recall Section 3.1). The value of tensile strength of some non-hybrid samples such as BC-5/0 and BC-0/5 is high, which is due to mechanical restraint to the easy sliding of polymer chains over one another. Furthermore, CaCO_3_ and bentonite nano particles are hard particles having a higher modulus than the virgin HDPE matrix. The degree of restrains to chain sliding increases as a result of the repulsive potential to the matrix which is closer in the vicinity of a hard particle. This entanglement of chains with filler particles has possibly majorly contributed to perform well as compared to the virgin polymer in the non-hybrid samples when the collective wt % of bentonite and CaCO_3_ in the sample are as low as 5% [33]. It is comprehensive now that this phenomenon can be effective when there are not enough filler particles to undergo agglomeration; however, the excessive loading of the fillers adversely affects the properties. As seen in Figure 7a,b, the filler particles agglomerates possibly have acted as “stony pulled out” particles during the test, causing further deterioration of strength, even more than the virgin polymer.

**Figure 8:**
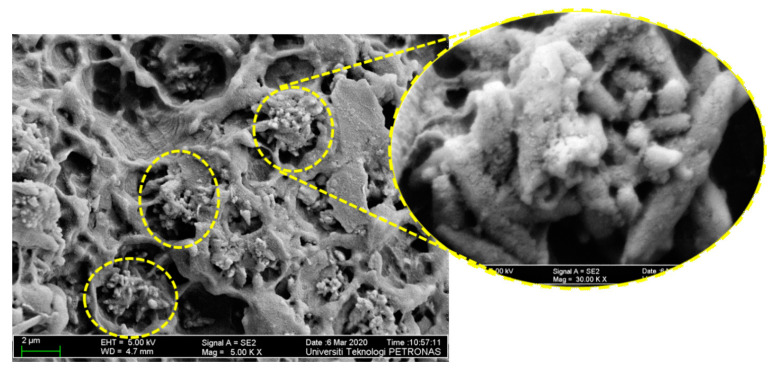
Field Emmision Scanning Electron Microscopic (FESEM) image of the hybrid sample BC-5/5 displaying the encapsulation of CaCO_3_ by bentonite.

**Figure 9:**
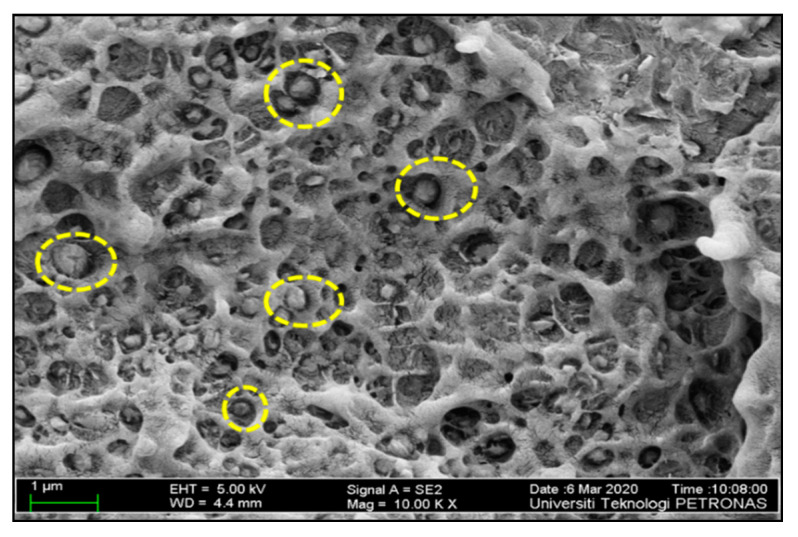
CaCO_3_ filler particles distributed separately with no encapsulation.

Furthermore, a new revelation of two distinct types of distribution filler particles in the HDPE matrix is discovered in FESEM analysis. The distinctive type of distribution is also reported in the work of Moghbelli et al. [14]. The filler particles in hybrid samples dominated by the contents of bentonite can be seen to undergo distribution “jointly” as compared to the separate distribution of particles in the samples of GA. This difference can be viewed by comparing Figure 8 and Figure 9. Several authors reported the effective role of nano clays in affecting the distribution of filler particle positively [34,35,36], which agrees with this explanation.

**Figure 10:**
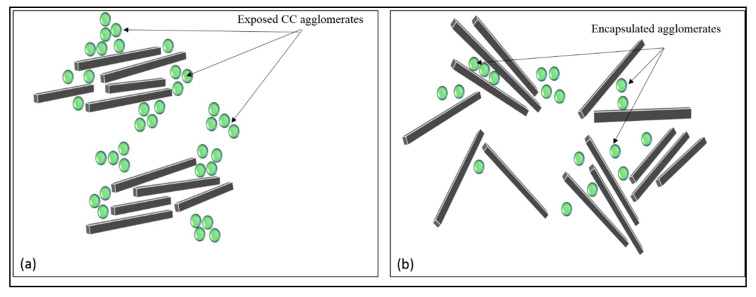
Schematic representation of the physio-mechanical phenomenon of the strengthening of composite (**a**) agglomerate particles distributed separately, (**b**) bentonite clay and CaCO_3_ particles distributed jointly, rendering the encapsulation of CaCO_3_ particles.

The work of wang et al. [17] showed that, during fabrication and tensile testing, the clay particles have a likelihood chance under the external shear of melt compounding to “open up”. The aggregate of clay layers was partially or fully dismantled under the effect of shear and the CaCO_3_ particles is encapsulated by the dismantled clay particles in this process. This combination of the “opening” and “encapsulation” of clay reduced the exposure of CaCO_3_ particles, thus reducing the incompatibility of CaCO_3_ to minor. These encapsulated CaCO_3_ particles are held up together by interlocking, as viewed at much higher magnification (30 k) of Figure 8. The interlocking/encapsulation of CaCO_3_ filler particles is shown by the FESEM image in Figure 8. It is analogous to the physio-mechanical phenomenon proposed in Figure 10. As clearly shown, the clay layers have separated from one another owing to higher bonding forces with the matrix as compared to the forces between one another [17]. Studies term this physio-mechanical phenomenon as the “adsorption” ability of nano clay towards filler particles residing alongside clay particles in a polymer matrix. The affinity of filler particles, described as being under the effect of different forces, also clearly explains the interlocking of CaCO_3_ particles by bentonite nano clay [37]. Therefore, the microstructural layout of bentonite can be considered for generating certain morphology to justify the preferential application of bentonite as a compatibilizers for a fine morphology.

Additionally, by doing so, the adverse effects of surfactants can be reduced by providing no chance of extra uncontrolled coating [13] and extra weak interphases [12]. Moreover, certain studies also dictate the use of bentonite as a solid compatibilizer because mechanical properties were reported to be adversely affected when compatibilizer was applied as compared to the sample without compatibilizers [14].

Given these points, when the content of bentonite is more, it adequately encapsulated CaCO_3_ particles, minimizing the exposure of CaCO_3_ particles and the distribution of CaCO_3_ particles. Such particles are enhanced by interlocking them separately, as displayed in Figure 8. Moreover, similar FESEM images of clay particles possessing the same features are already presented in the work of Salam et al. [38], which discussed the processing of different clay particles.

#### 3.5.2. Fractographic Analysis

**Figure 11:**
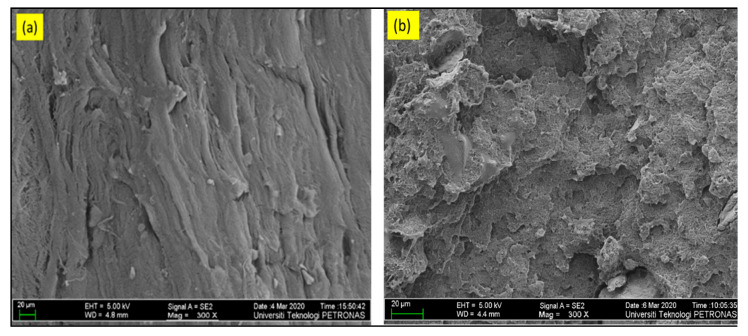
FESEM of cryofractured samples at a lower magnification (300×): (**a**) brittle fracture in BC-0/5 and (**b**) ductile fracture in BC-5/5.

Fractography of cryofracture samples can come up with useful knowledge about the final failure of the composite complementing the tensile test. Fractography of the samples is consistent with the morphological interpretation, i.e., clay-rich samples were identified with the pull out of resin as a result of the preferable stretching phenomenon in ductile fracture, as opposed to sharp and clear fracture of brittle failure [30]. Referring to Figure 11, the samples undergoing brittle fracture displayed fractographic patterns (at lower magnification 300×), as shown in Figure 11a. It looks as though easy and non-forceful exertion on the filler particles during crack propagation took place, while, to the lift in Figure 11b, scales appeared, indicating the marks left behind when the crack front propagates against well-bonded particles.

It can be established that samples undergoing the fracture, as shown in Figure 11b, have a great potential for the toughening of HDPE. These scale-like formations in fractograph are similar for all samples that were dominated by the content of bentonite.

**Figure 12:**
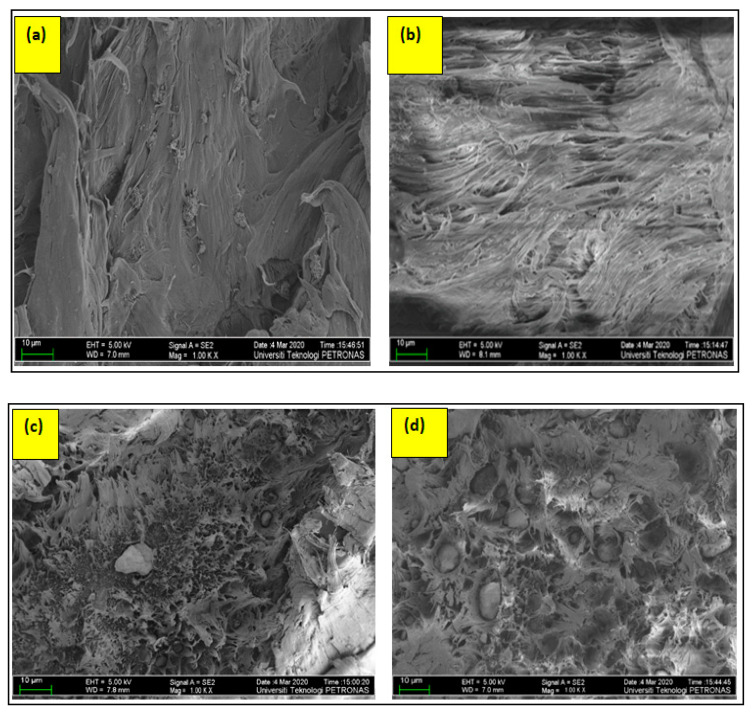

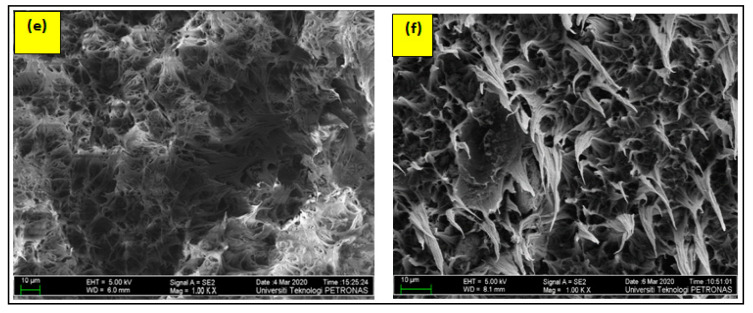
FESEM images of cryofractured samples (**a**) BC-0/5, (**b**) BC-5/15, (**c**)BC/10/10, (**d**) BC-15/15, (**e**) BC-15/5, (**f**) BC-5/5.

The direction of crack propagation is in the plane outwards from the page. The direction of the plane is towards the reader in Figure 12. It is noticeable that the rough patterns increases as we compare images from Figure 12a–f. The roughness of any particular fractograph in Figure 12 indicates the amount of force exerted for the propagation of cracks. A closer look at Figure 12c,d reveals the presence of some aggregate particles with cavities around the filler particles. It shows that, in case of the excessive loading of Clay in BC-15/15 and BC-10/10, there is always a chance of poor dispersion, as shown in the form of aggregates. Moreover, these large aggregates had, virtually, sustained break-up during processing and remained present in the blend [17]. Reflecting on the values of the tensile test, the anomalous behavior can be attributed to matrix-filler debonding at the interfaces, which can possibly be the reason for a mild toughness of both samples, as explained by their higher values of elongation [39].

Moreover, Figure 12f displays very rough patterns, dictating the justification for a ductile and the toughest response of the sample. The crack has likely followed a tortuous path of progression, first because there the clay particles were properly bonded to the matrix, and, secondly, the clay layers lying perpendicular to the crack front have possibly affected the path of crack propagation [17]. The polymer material can be seen to be pulled extensively with no visible filler particles entrapped or agglomerated. Such a pattern can also be noticed in Figure 12e, which is BC-15/5, but there is less pull of materials. All the stress-softened samples are more or less the same with the characteristic feature of rough patterns on Fractography. On the other hand, 12a displays a very smooth Fractography with no noticeable pulled out resin. To the lift in 12b, some seriations can be seen in BC-5/15. This is the only sample in GA having higher values of elongation, as comparable to the stress-softened samples in GA. It can be concluded that debonding at the polymer/filler interface induced shear yielding in the matrix and some amount of energy was absorbed during the fracture, making it somehow a tougher formulation [13].

The characteristic features of longer and more irregular striations in 12e and 12f apprise the story of the typical ductile fracture. The dismantling behavior of clay layers rendered the ductile fractography, as explained by the physio-mechanical phenomenon in Figure 10. Due to the re-orientation of clay layers, the pre-dominant process of toughening is crack deflection for highly ductile samples, while the cavities around the aggregates in Figure 12c,d suggest that, predominantly, the mild toughness is due to the polymer filler debonding.

**Figure 13:**
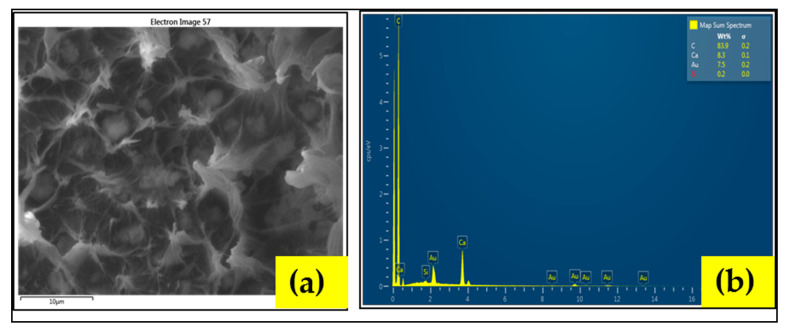
Elemental phase spectra (**a**) SEM of the cross section of the fracture surface (**b**) corresponding to the energy-dispersive X-ray spectroscopy (EDS) spectra of the cross section.

To verify, the ductile (toughening) behaviour without application of surfactants and the elemental analysis of the cross section of the fracture surface are performed via EDS (energy-dispersive X-ray spectroscopy) analysis, as shown in Figure 13. It can be seen that there is no traces of any extra surfactant chemicals. The peaks of Au, Si, Ca, C can be seen as the combination of carbon chains, CaCO_3_, and silicon components of nano clay. The peaks obtained on the surface of fracture also complements the EDS mapping performed for dispersion and distribution of the composite (recall Figure 2).

It can be concluded that the comparative weight % of fillers has affected the mode of fracture. The fractograph having rough patterns is the identification of energy exerted during crack propagation. The toughness induced by encapsulation is achieved without application of surfactant and compatibilizer.

## 4. Conclusions

This study provides a deep insight into the microstructural features of bentonite nanoclay, acting as a solid compatibilizer for HDPE polymer. The achieved objective of high strength is due to the chain entanglement phenomenon for samples with lower values of filler (5–10 wt %), while high strength for excessively loaded samples (10–25 wt %) is achieved first by a higher compatible nature of bentonite nano clay, and, secondly, by encapsulating CaCO_3_ particles during the opening up of clay layers. Moreover, the high value of elongation is due to HDPE-bentonite debonding during the later stages of tensile test.

The key points concluded from our experimental results are:

1. Sufficient strength transfer between HDPE-bentonite and CaCO_3_ can be achieved without the application of surfactants.
2. The values of elongation of the samples undergoing stress softening are higher while Young’s modulus is compromised in case of stress softening.
3. Stress-softened samples are identified with more toughness, having higher values of elongation, and rougher patterns were noted in fractography thus more ductility.
4. It is evident from EDS mapping and elemental characterization that inhomogeneity was reduced by the compatibilization effect of bentonite nano clay, but this is limited to the case when the content of bentonite exceeded that of CaCO_3_ or in some cases when an equal amount of bentonite and CaCO_3_ was used.
5. Similarly, CaCO_3_ nano particles were encapsulated when the bentonite nano clay dominated the samples. In other cases, there was not enough bentonite to perform this encapsulation and properties were not enhanced.

The process of obtaining low HDPE-bentonite and CaCO_3_ adhesion (to favor debonding for toughness) and, at the same time, adequate bonding between HDPE-bentonite and CaCO_3_ for high strength and stable morphology is contradictory in nature. However, the finer compatibilization of bentonite nano clay achieved better low particle–matrix adhesion. In the absence of surfactant, debonding was allowed only in the last stages of fracture giving out higher values of ductility and toughness. Having discussed all aspects, we can conclude that reinforcing HDPE does not need the application of surfactants and compatibilizers in all cases. The selection of nano fillers on the base of surface properties can be seen as a substitute for improving properties of HDPE. Therefore, this expands the range of applicability of HDPE and rigid fillers, specifically for pseudo-ductile applications.

## Figures and Tables

**Table 1:** Summary of mechanical properties resulting from the tensile testing of group A (GA).

Sample	Tensile Strength (MPa)	Elastic Limit (MPa)	E (MPa)	Elongation at Break (mm)
BC-0/0	19.06 ± 0.48	10.65 ± 2.31	596 ± 58.43	17.91 ± 0.48
BC-0/5	23.06 ± 4.50	13.24 ± 5.60	788 ± 60.70	7.85 ± 5.6
BC-0/10	16.06 ± 3.60	12.669 ± 3.50	515 ± 80.90	2.51 ± 1.5
BC-0/15	16.46 ± 6.40	11.65 ± 4.70	331 ± 70.39	8.73 ± 3.80
BC-5/10	18.31 ± 3.80	15.99 ± 5.90	372 ± 30.51	6.40 ±4.87
BC-5/15	20.37 ± 7.50	12.63 ± 2.31	937 ± 42.39	10.16 ± 4.50
BC-10/15	15.36 ± 6.30	15.35 ± 3.50	498 ± 56.51	4.50 ± 1.40

**Table 2:** Summary of Mechanical Properties resulting from the tensile testing of group B (GB).

Sample	Tensile Strength (MPa)	Elastic Limit (MPa)	E (MPa)	Elongation at Break (mm)
BC-0/0	19.06 ± 0.48	10.65 ± 2.31	596 ± 58.43	17.91 ± 0.48
BC-5/0	20 ± 1.44	12.97 ± 5.91	772 ± 59.58	4.11 ± 7.9
BC-10/0	17.28 ± 8.31	14.88 ± 3.21	308 ± 64.59	7.95 ± 6.5
BC-10/5	21.28 ± 5.40	12.53 ± 4.57	563 ± 80.90	9.9 ± 4.50
BC-15/0	16.75 ± 8.39	12.44 ± 6.51	363 ± 60.21	7.02 ± 6.50
BC-15/5	20.41± 7.49	16.10 ± 8.48	490 ± 30.32	10.76 ± 8.90
BC-15/10	20.09 ± 6.50	15.73 ± 7.77	552 ± 40.51	9.53 ± 3.80

**Table 3:** Summary of Mechanical Properties resulting from the tensile testing of group C (GC).

Sample	Tensile Strength (MPa)	Elastic Limit (MPa)	E (MPa)	Elongation at Break (mm)
BC-0/0	19.06 ± 0.48	10.65 ± 2.31	596 ± 58.43	17.91 ± 0.48
BC-5/5	21.61 ± 1.85	18.69 ± 5.60	448 ± 63.66	17.53 ± 8.9
BC-10/10	19.22 ± 7.86	12.58 ± 6.70	619 ± 75.78	5.77 ± 5.6
BC-15/15	17.89 ± 3.41	16.29 ± 8.90	345 ± 89.67	6.37 ± 4.3
